# Photosynthetic Versatility in the Genome of *Geitlerinema* sp. PCC 9228 (Formerly *Oscillatoria limnetica* ‘Solar Lake’), a Model Anoxygenic Photosynthetic Cyanobacterium

**DOI:** 10.3389/fmicb.2016.01546

**Published:** 2016-10-13

**Authors:** Sharon L. Grim, Gregory J. Dick

**Affiliations:** Department of Earth and Environmental Sciences, University of Michigan, Ann ArborMI, USA

**Keywords:** cyanobacteria, anoxygenic photosynthesis, great oxidation event, nitrogenase, photosystem II D1 protein, sulfide quinone reductase

## Abstract

Anoxygenic cyanobacteria that use sulfide as the electron donor for photosynthesis are a potentially influential but poorly constrained force on Earth’s biogeochemistry. Their versatile metabolism may have boosted primary production and nitrogen cycling in euxinic coastal margins in the Proterozoic. In addition, they represent a biological mechanism for limiting the accumulation of atmospheric oxygen, especially before the Great Oxidation Event and in the low-oxygen conditions of the Proterozoic. In this study, we describe the draft genome sequence of *Geitlerinema* sp. PCC 9228, formerly *Oscillatoria limnetica* ‘Solar Lake’, a mat-forming diazotrophic cyanobacterium that can switch between oxygenic photosynthesis and sulfide-based anoxygenic photosynthesis (AP). *Geitlerinema* possesses three variants of *psbA*, which encodes protein D1, a core component of the photosystem II reaction center. Phylogenetic analyses indicate that one variant is closely affiliated with cyanobacterial *psbA* genes that code for a D1 protein used for oxygen-sensitive processes. Another version is phylogenetically similar to cyanobacterial *psbA* genes that encode D1 proteins used under microaerobic conditions, and the third variant may be cued to high light and/or elevated oxygen concentrations. *Geitlerinema* has the canonical gene for sulfide quinone reductase (SQR) used in cyanobacterial AP and a putative transcriptional regulatory gene in the same operon. Another operon with a second, distinct *sqr* and regulatory gene is present, and is phylogenetically related to *sqr* genes used for high sulfide concentrations. The genome has a comprehensive *nif* gene suite for nitrogen fixation, supporting previous observations of nitrogenase activity. *Geitlerinema* possesses a bidirectional hydrogenase rather than the uptake hydrogenase typically used by cyanobacteria in diazotrophy. Overall, the genome sequence of *Geitlerinema* sp. PCC 9228 highlights potential cyanobacterial strategies to cope with fluctuating redox gradients and nitrogen availability that occur in benthic mats over a diel cycle. Such dynamic geochemical conditions likely also challenged Proterozoic cyanobacteria, modulating oxygen production. The genetic repertoire that underpins flexible oxygenic/anoxygenic photosynthesis in cyanobacteria provides a foundation to explore the regulation, evolutionary context, and biogeochemical implications of these co-occurring metabolisms in Earth history.

## Introduction

With a long evolutionary history and wide ecological success, cyanobacteria are pivotal mediators of Earth’s geochemical cycles, most notably through oxygenic photosynthesis (OP). This metabolism emerged early in cyanobacteria (Blankenship, 2010; Farquhar et al., 2010), and oxygenic cyanobacteria that colonized newly formed continental margins and shallow seas in the Archean (Reddy and Evans, 2009; Lalonde and Konhauser, 2015) were the leading mechanism for partial oxygenation of these shallow regions (Planavsky et al., 2014; Satkoski et al., 2015). Although, cyanobacterial OP is the major biological force behind Earth’s oxygenation (Blankenship, 2010; Crowe et al., 2013; Planavsky et al., 2014), the biological and geological processes that influence cyanobacterial oxygen production and thus underpin Earth’s oxygenation are still under debate (Lyons et al., 2014).

The balance of oxygenic and anoxygenic photosynthesis (AP) has been proposed as a biological mechanism to explain the delay and variability in oxygenation (Johnston et al., 2009). This includes competition between AP bacteria and OP cyanobacteria, interactions between different cyanobacterial groups with varying degrees of AP or OP specialization, as well as cellular regulation of the photosynthetic modes within metabolically flexible cyanobacterial groups. Atmospheric oxygenation is considered key evidence for OP, but the emergence and development of AP in cyanobacteria is less clear. Assuming an early evolution of photosynthetic flexibility in cyanobacteria, AP cyanobacteria may have had an important role in sustaining ancient ecosystems from the end of the Archean through the Proterozoic, especially in times of global and local variability of O_2_, fixed nitrogen, and alternative electron donors for photosynthesis such as H_2_S and Fe(II) (Canfield, 1998; Scott et al., 2008; Lyons et al., 2014; Sperling et al., 2015).

Molecular innovations equipped cyanobacteria for OP and nitrogen fixation in a dynamic environment. The development of OP required the linkage of two light-driven reaction centers: photosystem II, which produces oxygen from the oxidation of water; and photosystem I, which transfers electrons from plastoquinone to ferredoxin (Blankenship, 2002). This coupled system capitalized on the wide availability of H_2_O compared to more limited supply of electron donors for AP such as Mn(II), Fe(II), and H_2_S (Blankenship, 2002). Homologous proteins D1 and D2, encoded by the *psbA* and *psbD* genes, form the core of PSII and anchor the water oxidizing complex (Ferreira et al., 2004; Fischer et al., 2015). Modern cyanobacteria have multiple versions of *psbA* to cope with different oxygen levels and light regimes (Mohamed et al., 1993). Cyanobacterial adaptations to an aerobic lifestyle are also reflected in the *nif* genes for nitrogen fixation, which initially emerged in methanogens in an anoxic environment (Raymond et al., 2004; Boyd et al., 2011). Reflecting increasing oxygen levels, cyanobacterial genomes lost and recruited nitrogenase (*nif*)-related genes, and shifted expression patterns and regulation that enabled nitrogen fixation in an oxic world (Boyd et al., 2015).

Studies of modern cyanobacteria have provided insights into how sulfide may have modulated the balance of OP and AP in ancient ecosystems. The influence of sulfide on cyanobacterial photosynthesis ranges from complete inhibition at even low levels of H_2_S to resilience or resistance to sulfide toxicity. In some cyanobacteria, sulfide exposure may induce AP (Cohen et al., 1986; Miller and Bebout, 2004), which does not include PSII and thus does not produce O_2_. Instead, the sulfide quinone reductase (SQR, coded for by the *sqr* gene) oxidizes sulfide to sulfur and transfers electrons to PSI (Arieli et al., 1994; Theissen et al., 2003). Such AP cyanobacteria have been documented in hypersaline lakes (Cohen et al., 1975a), sinkholes (Voorhies et al., 2012), and sulfidic springs (Chaudhary et al., 2009; Bühring et al., 2011; Klatt et al., 2016). Studied cyanobacteria have different mechanisms for the transition between OP and AP, such as protein synthesis (Oren and Padan, 1978), a dependence on light quantity and spectrum, and kinetics and affinities between enzymes and quinones (Klatt et al., 2015a). The physiology of AP cyanobacteria and their potential importance in modern and ancient ecosystems have been previously explored, yet the genomic basis for this flexible metabolism is poorly understood.

*Geitlerinema* sp. PCC 9228, formerly *Oscillatoria limnetica* ‘Solar Lake’, is a model anoxygenic photosynthetic cyanobacterium. The organism was cultured from the low-light sulfidic hypolimnion of Solar Lake, below a layer of green and purple sulfur bacteria (Cohen et al., 1975b). Filamentous cyanobacteria such as *Geitlerinema* are rare in oxic and well-illuminated surface waters, and most numerous in the euxinic hypolimnion at which they receive a fraction of surface irradiance (Cohen et al., 1977b). In laboratory experiments at light intensities similar to or higher than *in situ* levels, *Geitlerinema* performs OP, but transitions fully to sulfide-based AP at micromolar concentrations of sulfide (Cohen et al., 1986). Under sulfidic conditions, *Geitlerinema* can also fix nitrogen and produce hydrogen (Belkin and Padan, 1978; Belkin et al., 1982). Its SQR has been isolated (Arieli et al., 1994), sequenced (Bronstein et al., 2000), and phylogenetically characterized (Pham et al., 2008; Marcia et al., 2010a; Gregersen et al., 2011). *Geitlerinema* is a model organism for studying the physiology of flexible AP/OP, diazotrophic cyanobacteria and their influence on modern and ancient systems. In this study, we analyzed a draft genome of *Geitlerinema* and characterized the genes related to nitrogen fixation, AP, and OP. These results provide a genomic foundation for metabolic flexibility in response to varying sulfide, oxygen, and light levels that was observed in previous physiology studies (Belkin and Padan, 1978; Belkin et al., 1982; Cohen et al., 1986; Shahak et al., 1987).

## Materials and Methods

### Culturing and Sequencing

The original strain was isolated from the sulfidic water column of Solar Lake, Israel (Cohen et al., 1975b), and was kindly provided by A. Oren for culturing. A monoalgal culture was grown in modified Chu’s 11 in Turks Island Salts medium at room temperature (average 22.0°C) and ambient light in a 125 mL Erhlenmeyer flask. We extracted whole community DNA using the MPBio FastDNA SpinKit and Fastprep-24 Bead Beater (MP Biomedicals, Solon, OH, USA) following the default protocol, except that 0.3 g of beads were used for bead beating. DNA was quantified using Quant-IT PicoGreen (Invitrogen, Grand Island, NY, USA) and submitted to the University of Michigan DNA Sequencing Core for library preparation and Illumina HiSeq 2 × 100 bp paired-end sequencing.

### Assembly

Using wrappers provided at https://github.com/Geo-omics/scripts, reads were dereplicated with custom perl scripts, trimmed using Sickle (version 1.33) (Joshi and Fass, 2011), and assembled using IDBA-UD (version 1.1.1) (Peng et al., 2012) with the following parameters: –mink 65 –maxk 85 –step 10 –pre_correction. Tetranucleotide frequency was used to bin scaffolds by emergent self-organizing maps (Dick et al., 2009), with a minimum contig length of 500 bp and a window size of 10,000 bp (**Supplementary Figure S1**). The cyanobacterial bin of scaffolds >1000 bp in length was submitted to the Integrated Microbial Genomes Expert Review (IMG-ER) automated pipeline from Joint Genomes Institute (JGI) for annotation of genes and pathways (IMG accession number: 2660238729; Supplementary Table S1). Raw reads, assembled scaffolds, and gene annotations were submitted to NCBI (project: PRJNA302164).

### Phylogenetic Analysis

Phylogenetic analyses of genes of interest (**Table 1**) was performed with maximum likelihood and the PROTGAMMAGTR algorithm in RAxML 8.1.15 and bootstrapped 1000 times (Stamatakis, 2014). *psbA. sqr*, and *nifHDK* gene phylogenies included 44 cyanobacterial isolate genomes and genomic bins from Hot Lake and Middle Island Sinkhole cyanobacterial mat metagenomes (Cole et al., 2014; Voorhies et al., 2016; Supplementary Table S2). Each of these cyanobacterial genome has at least one *sqr* gene, and 30 of the 44 genomes have a *nifHDK* gene set. *sqr* and *nifHDK* genes from *Sulfuricurvum kujiense* YK-1 DSM 16994, and other bacterial *sqr* genes (Supplementary Table S3) were included for context. Translated genes (amino acid sequences) were aligned with clustal-omega (version 1.2.0) (Sievers et al., 2011). Alignments for *nifH. nifD*, and *nifK* were concatenated, and the concatenated alignment was used for analysis. *Halothece halophytica chlLNB* genes were used to root the *nifHDK* tree (Boyd et al., 2015). Flavocytochrome c:sulfide dehydrogenase (FCSD) genes formed an outgroup for the *sqr* tree (Marcia et al., 2010a). The most divergent *psbA* from *Gloeobacter kilaueensis* JS-1 was used as an outgroup for the *psbA* tree (Cardona et al., 2015). Phylogenetic trees were drawn with FigTree version 1.4.2 (Rambaut, 2012).

**Table 1:** Integrated Microbial Genomes (IMG) accession numbers of genome and select genes of interest of *Geitlerinema* sp. PCC 9228.

Description	IMG ID
Genome ID	2660238729
*nifH*	2663545465
*nifD*	2663545468
*nifK*	2663545469
*psbA1*	2663545011
*psbA2*	2663544218
*psbA3*	2663548115
*sqr1*	2663545736
*sqr2*	2663547046


## Results

### Overview

The 4.77 Mb draft genome of *Geitlerinema* sp. PCC 9228 (henceforth “*Geitlerinema*”) contains 3,969 protein coding genes on 195 scaffolds. Coverage is on average 905x across the genome. The genome has 100% of universally conserved bacterial genes expected to be present (Raes et al., 2007; Alneberg et al., 2014).

### Carbon Metabolism

**FIGURE 1:**
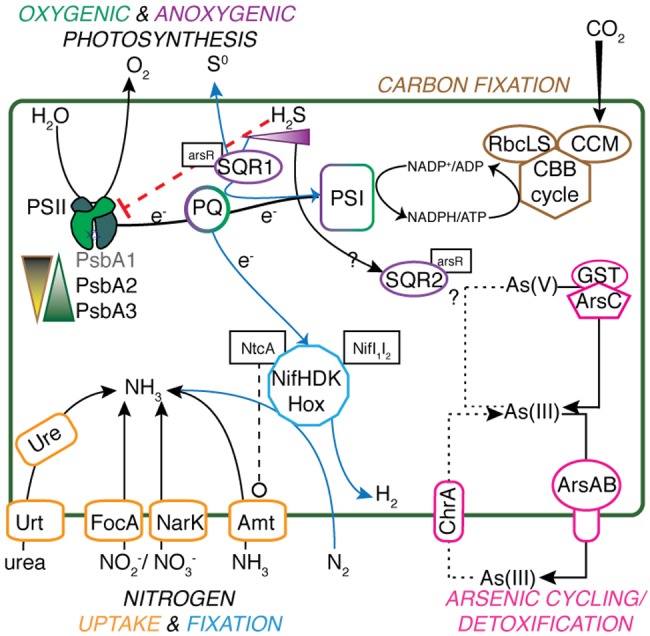
**Metabolic schematic of *Geitlerinema* sp. PCC 9228.** Genes for major processes [*psbA1-3, sqr1, ccm, rbcLS, nifHDK, hox, arsABC, ure, urt, focA, narK, amt*, glutathione *S*-transferase (GST)], and associated regulatory genes (*arsR, ntcA, nifI_1_I_2_*) are presented. Suppression of functions is indicated with red dashed lines ending in a flat line. Other interactions are indicated with black dashed lines ending with open circles. Transfer and production of key reactants and products are indicated with solid lines. Putative arsenite import is indicated with a dotted line, and anoxygenic photosynthesis-related processes have a solid blue line.

*Geitlerinema* has key genes coding for proteins involved in photosynthetic and respiratory electron flow, including photosystem II (*psbADBCEFO*), succinate dehydrogenase (*sdhABC*), type-1 NADPH dehydrogenase (*ndhA-M*), cytochrome b6f (*petADBCEJ*), photosystem I (*psaABCDEFK*), and cytochrome c oxidation (*coxABC*) (Supplementary Table S1; Mulkidjanian et al., 2006). It has genes for the Calvin-Benson-Bassham cycle, carbon dioxide concentrating proteins (*ccmK_1_K_2_MN*), and Rubisco large and small subunits (*rbcLS*) for carbon fixation (**Figure 1**). Superoxide dismutase genes (*sodC* and *sodN*) to cope with superoxide formation during photosynthesis and aerobic respiration are also present in the genome. In ancestral cyanobacteria, these enzymes would have been critical for defense against increased production of reactive oxygen species alongside increasing O_2_ fluxes (Blank and Sánchez-Baracaldo, 2010; Fischer et al., 2016).

*Geitlerinema* has genes encoding a complete tricarboxylic acid (TCA) cycle, including 2-oxoglutarate dehydrogenase and succinyl CoA synthase to link synthesis of 2-oxoglutarate through succinyl-coA with succinate (Steinhauser et al., 2012). The genome also has the genes for acetolactate synthases and succinate-semialdehyde dehydrogenase to interconvert 2-OG and succinate through succinic semialdehyde in an alternative closure to the TCA cycle (Zhang and Bryant, 2011). Though it has *shc*, encoding for squalene-hopene cyclase, *Geitlerinema* lacks the *hpnP* gene for hopanoid methylation (Ricci et al., 2015). 2-methylhopanes, derived from 2-methylhopanoids, have been used as a bacterial biomarker in the geologic record (Summons and Lincoln, 2012). *Geitlerinema* has genes for acyl-ACP reductase and fatty aldehyde decarbonylase, key enzymes in an alkane biosynthesis pathway unique to cyanobacteria (Schirmer et al., 2010; Coates et al., 2014).

In the chlorophyll synthesis pathway, we identified both the aerobic oxidative ester cyclase *chlE* common to all cyanobacteria (Mulkidjanian et al., 2006), and the oxygen independent ester cyclase *bchE*. Functional *bchE* is common in anoxygenic phototrophic bacteria such as green sulfur bacteria and heliobacteria (Sousa et al., 2013), and is only rarely present in cyanobacteria, such as *Synechocystis* sp. PCC 6803 (Minamizaki et al., 2008). *Geitlerienema* has the light-dependent NADPH-protochlorophyllide oxidoreductase that produces chlorophyllide in the penultimate step of the pathway. This gene originated in cyanobacteria and is limited to cyanobacteria and phototrophic eukaryotes (Blankenship, 2002; Yang and Cheng, 2004). However, the genome lacks the light-independent oxidoreductase *chlLNB*, which allows for chlorophyll synthesis in the dark and is observed in all photosynthetic phyla (Blankenship, 2002). Due to their homology, *Geitlerinema*’s genes for *nifHDK* are the closest matches to *H. halophytica’s chlLNB*. Coverage estimates for *nifHDK* genes are consistent with the rest of the genome, indicating there was no mis-assembly of *chlLNB* reads into *nifHDK* genes.

### Nitrogen Metabolism

The cyanobacterial genome has genes for a variety of pathways of nitrogen acquisition, including an ammonia transporter gene *amt*, cyanate lyase *cynS*, nitrate/nitrite transporters *narK* and *focA*, and nitrate assimilation-related genes nitrate and nitrite reductases *nirA. nirC*, and *narB* (**Figure 1**). The cyanobacterium also has urea transporters (*urtABCD*), urease genes (*ureABCDFG*), and genes for transport of neutral, branched, and polar amino acids.

**FIGURE 2:**
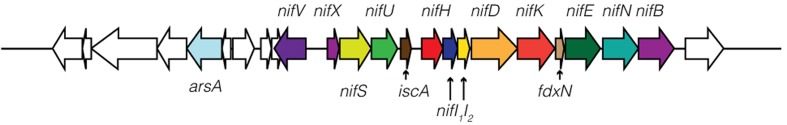
**Schematic of nitrogenase genes in *Geitlerinema* sp. PCC 9228.** *nifXSU, iscA, nifHI_1_I_2_DK, fdxN*, and *nifENB* are arranged in an apparent operon. *nifV* and a regulatory arsenic-related gene *arsA* are located upstream of the *nifHDK* operon.

*Geitlerinema* has a comprehensive operon for nitrogen fixation (*nifVXSU, nifHDKEB*; Raymond et al., 2004). Additional nitrogenase-related proteins are located on the same contig (*iscA. nifI_1_I_2_*, ferredoxin, *nifN*; **Figure 2**; Boyd et al., 2015). *iscA* is commonly observed in aerobic diazotrophs, and its recruitment into the genome is linked to a transition to aerobic lifestyle (Boyd et al., 2015). The cyanobacterium has *glnB*, a member of the PII signal transduction protein family that regulates nitrogen-related proteins, and *ntcA*, which controls expression of *glnB* (Forchhammer, 2004). The *nifI_1_I_2_* gene is also a member of the PII protein family, but is characteristic of diazotrophic anaerobes that regulate their nitrogenase activity post-translation, such as *Desulfovibirio* and *Clostridium* (Boyd et al., 2015).

Phylogenetic analysis shows that *nifHDK* genes from *Geitlerinema* clusters with those from two cultured cyanobacterial genomes (*Pleurocapsa* sp. 7327, *Microcoleus chthonoplastes*) and a cyanobacterial genome-from-meta genomic bin (*Phormidium* OSCR; bootstrap = 100; **Supplementary Figure S2**). Like *Geitlerinema*, their nitrogenase operons also hold *nifI_1_I_2_. Geitlerinema* has a bidirectional NiFe hydrogenase gene set (*hoxEFUYH* and *hoxW*) with its transcriptional regulator (*lexA*) and hydrogenase maturation proteins (*hypBAEDC*) (**Figure 1**). Unlike *Geitlerinema*, typical nitrogen-fixing cyanobacteria have an uptake hydrogenase (*hupSL*) to consume H_2_ produced in nitrogen fixation. *hupSL* is under similar transcriptional regulation as nitrogen acquisition genes like dinitrogenase, making expression of *hupSL* dependent on nitrogen limitation (Tamagnini et al., 2007). In contrast, the bidirectional hydrogenase can be present in both diazotrophic and non-diazotrophic cyanobacteria and is expressed under more diverse conditions. It may, for instance, be used in fermentation or to direct electrons during photosynthesis (Tamagnini et al., 2007). *Geitlerinema* produces hydrogen during sulfide-dependent AP in the presence of bioavailable nitrogen, as well as in the absence of CO_2_, suggesting the nitrogenase-independent *hox* gene set may be responsible for hydrogen evolution (Belkin and Padan, 1978; Belkin et al., 1982).

### Photosystem II Assembly

**FIGURE 3:**
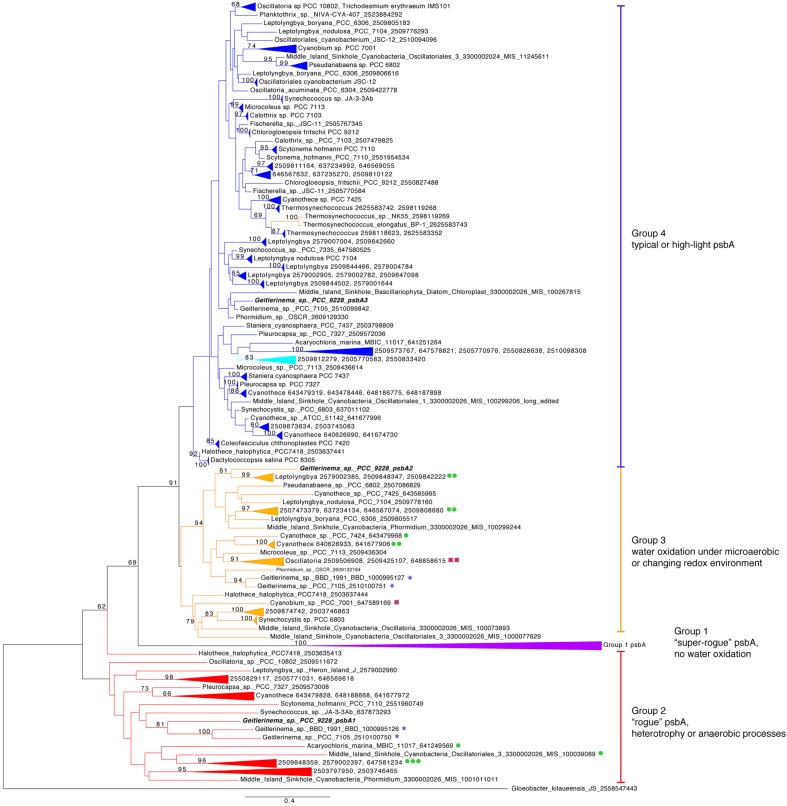
**Phylogenetic tree of *psbA*.** Genes are colored by groups 1–4 modeled after (Cardona et al., 2015). The outgroup is a *Gloeobacter kilaueensis JS psbA*, which is most similar to *psbD* and is unable to oxidize water (Cardona et al., 2015). Purple indicates group 1, red genes belong to group 2, orange genes are members of group 3, and blue standard *psbA*s belong to group 4. Genetic proximity to *sqr* types I (green circle), II (maroon squares), and VI (blue stars) *sqr* genes are indicated. Fourteen *psbA* genes are five or fewer genes from *sqr*, and the largest gap is 19 genes. *Geitlerinema* sp. PCC 9228 has members of groups 2–4.

The core proteins in photosystem II are encoded by *psbA* and *psbD* (Blankenship, 2002). *Geitlerinema* has one version of *psbD* and three versions of the *psbA* gene (**Figure 1**). The standard *psbA*, hereafter referred to as *psbA3*, is in a large clade of typical oxygenic *psbA* designated “group 4” after (Cardona et al., 2015) (**Figure 3**). All but one of the isolate genomes in this analysis have at least one copy of this form of *psbA*, which is used in OP in aerobic conditions (Supplementary Table S2). Coverage estimates and paired-end information suggest that *Geitlerinema* has two copies of *psbA3*. It is the sole gene on its contig, and pairs of reads that map to the ends of the *psbA3* match to the ends or beginnings of four other contigs. Those portions have 100% identity to the ∼80 bp beginning and end of *psbA3*. The De Bruijn graph-based assembly algorithm used in this analysis frequently prematurely assembles identical copies of genes (Nagarajan and Pop, 2013). The approach accurately assembled the two copies of *psbA3*, but could not automatically bridge the copies.

One additional version of *psbA*, henceforth referred to as *psbA1*, has 85% sequence identity to *psbA3* (blastx). *psbA1* is located in a different operon but on the same scaffold near *ntcA* and photosynthetic subunits *psbN* and *psbH. psbA1* is in a well-supported clade with genes from *Geitlerinema* sp. PCC 7105 (2510100750) and *Geitlerinema* sp. BBD 1991 (BBD_1000995126; bootstrap = 81; **Figure 3**). This gene is also in a larger “group 2” (Cardona et al., 2015) that includes *psbA* genes from cyanobacterial genomic bins (MIS_1001011011, MIS_100039089) sourced from a low-oxygen cyanobacterial mat in the Middle Island Sinkhole (Voorhies et al., 2016). Group 2 genes encode D1 proteins used under growth conditions that do not favor water oxidation (Cardona et al., 2015), such as heterotrophy in the dark (Park et al., 2013), photosynthetic electron transport inhibition (Kiss et al., 2012), or oxygen-sensitive processes such as nitrogen fixation (Toepel et al., 2008; Wegener et al., 2015). Finally, the third variant, *psbA2*, is 59–88% similar to *psbA1* (discontinuously; tblastx), and 90% similar to *psbA3* (over 99% of their lengths; blastx). *psbA2* is in a clade with genes from a Middle Island Sinkhole cyanobacterium (MIS_100299244), *Geitlerinema* sp. BBD1991 (BBD_1000995127) and *Phormidium* sp. OSCR (2609132164; bootstrap = 94). Proteins from these “group 3” *psbA* genes (Cardona et al., 2015) are expressed under microaerobic conditions, with modified electron transfer to cope with the changing redox environment (**Figure 1**; Sicora et al., 2004, 2009; Sugiura et al., 2012).

### Sulfide Oxidation

**FIGURE 4:**
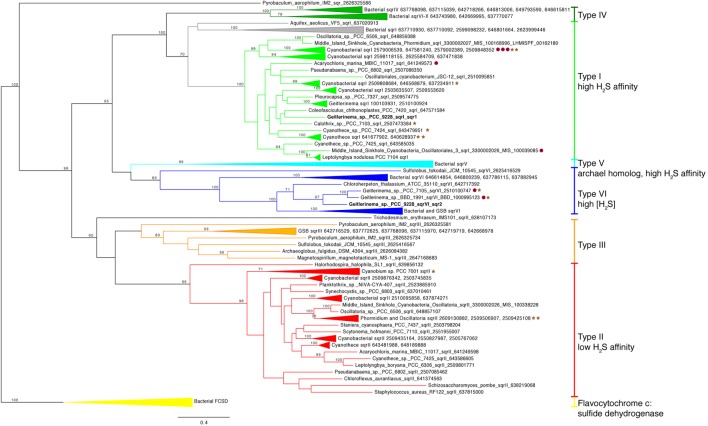
**Phylogenetic tree of sulfide quinone reductase (*sqr*).** Genes are colored by types I–VI modeled after (Marcia et al., 2010a). Bacterial FCSD (yellow) includes representatives of flavocytochrome c:sulfide dehydrogenase. Green sulfur bacteria have *sqr* belonging to types III (orange), V (light blue), IV (dark green), and VI (blue). Cyanobacterial *sqr* are limited to types I (light green), II (red), and VI. Proximity to *psbA* versions on the same contig (see **Figure 3**) are indicated with red circles (group 2) and brown stars (group 3). No *sqr* genes in this analysis were genetically proximal to groups 1 or 4 *psbA* genes. *Geitlerinema* sp. PCC 9228 has types VI and I *sqr*.

Two copies of the gene for sulfide quinone reductase, *sqr*, are in the genome of *Geitlerinema* (**Supplementary Figure S4**). This gene is involved in the oxidation of sulfide for detoxification or to harvest electrons for AP, such as in purple and green sulfur bacteria (Theissen et al., 2003; Marcia et al., 2009, 2010a; Gregersen et al., 2011). Each *sqr* is located upstream of arsenic resistance *arsR-*type genes, putatively involved in transcriptional regulation of *sqr* under conditions such as sulfide exposure (Nagy et al., 2014). One *sqr*, referred to as *sqr1* hereafter, matches the previously cloned and sequenced *sqr* gene of *Geitlerinema* (Bronstein et al., 2000). This enzyme mediates the reduction of plastoquinone and oxidation of sulfide in sulfide-dependent AP (Arieli et al., 1994). On the *sqr* phylogenetic tree, Geitlerinema’s canonical *sqr* groups with other cyanobacterial versions that are considered type I (Marcia et al., 2010a; Gregersen et al., 2011; bootstrap = 100; **Figure 4**; **Supplementary Figure S5**). Hydrogen sulfide affinities for cyanobacterial *sqr* in this cluster are high, with K_m_ in the micromolar range (Arieli et al., 1991; Bronstein et al., 2000). *Geitlerinema. Coleofasciculus* (formerly *Microcoleus*), *Halothece*, and proteobacterial members in this cluster have been shown to grow with this *sqr* on sulfide-induced AP (Oren and Padan, 1978; Cohen et al., 1986; Jørgensen et al., 1986; Schütz et al., 1999).

The second *sqr* gene, referred to as *sqr2* hereafter, is 25–50% identical over discontinuous fragments to the *sqr1* gene (tblastx). It is most similar (tblastx 48–63% identity to overlapping fragments that span the length of the gene) to that of *Chloroherpeton thalassium* ATCC35110, a green sulfur bacterium, with similar identity to the *sqr* genes of other sulfur oxidizing bacteria. *sqr2* clusters phylogenetically with two other cyanobacterial *sqr* [*Geitlerinema* sp. PCC 7105 and BBD 1991 (bootstrap = 97)], among a group of known type VI versions (bootstrap = 100; **Figure 4**; **Supplementary Figure S5**). They include *sqr* from thiotrophic proteobacteria (Marcia et al., 2010a) and green sulfur bacteria (Gregersen et al., 2011), including that of *Chlorobium tepidum* used in sulfide oxidation when sulfide exceeds 4 mM (Chan et al., 2009).

### Trace Metal Resistance

Arsenic resistance genes *arsB* (an arsenite efflux pump) and *arsC* (arsenate reductase; Slyemi and Bonnefoy, 2011; van Lis et al., 2013), and *arsR*, the putative regulatory protein of *sqr*, are in an *arsRBC* operon downstream of *sqr2*. Upstream of *sqr2* are genes encoding glutathione S-transferase and a multidrug efflux pump (**Supplementary Figure S4**). Another *arsB* is located downstream of *sqr1* but in a different operon. In combination with *arsB*, two arsenite-tranporting ATPases *arsA* genes are present in the genome, one located on the same scaffold as the nitrogenase gene suite and the other on the longest scaffold in the dataset. *Geitlerinema* does not have known arsenite oxidizing genes (*aioAB. arxA*) or respiratory arsenate reductase genes (*arrA*), based on gene annotation and BLAST searches against known genes (Hoeft et al., 2010; Slyemi and Bonnefoy, 2011). It has an annotated chromate transporter (*chrA*) on another scaffold that has 52% positive match (blastp) to a similarly annotated gene in *Synechocystis* sp. PCC6803 that functions as an arsenite uptake transporter for arsenite oxidation (Nagy et al., 2014; **Figure 1**). On another scaffold, a gene belonging to the DUF302 protein superfamily of unknown function is quite similar (75% positive, 56% identical blastp) to a *Synechocystis* gene in the *sqr1* plasmid operon, as well as to a potential arsenic oxidase gene in *Agrobacterium* (61% positive, 44% identical, blastp; Nagy et al., 2014). *Geitlerinema* also has a methyltransferase similar (84% positive, blastx) to the *arsM* gene of *Synechocystis* used in arsenite methylation for detoxification (Yin et al., 2011).

## Discussion

Previous investigations of cyanobacterial cultures capable of OP and sulfide-dependent AP targeted physiology, biochemistry, and limited genetic analyses (Cohen et al., 1986; Arieli et al., 1994; Bronstein et al., 2000; Miller and Bebout, 2004; Klatt et al., 2015a). However, the broader genetic characteristics of this cyanobacterial metabolic flexibility have not been thoroughly evaluated. In this study, we analyzed the genome of a model anoxygenic photosynthetic cyanobacterium, *Geitlerinema* sp. PCC 9228. The organism was isolated from a sulfidic, low-light environment, and numerous physiology studies have documented its ability to fix nitrogen, its high affinity for sulfide, and its oxygenic and anoxygenic photosynthetic capabilities (Cohen et al., 1975b, 1986; Belkin et al., 1982). We comprehensively evaluated the draft genome of *Geitlerinema* for key metabolic genes for oxygen, nitrogen, and carbon cycling, and provide the genetic evidence of its metabolic versatility.

### Photosynthesis Is Optimized for Light and Redox

Our results indicate that *Geitlerinema* sp. PCC 9228 and other sulfide-tolerant and/or sulfide-using cyanobacteria have multiple different types of *psbA* genes that are inferred to meet physiological and biochemical needs under changing light and redox conditions. The *psbA* gene encodes the D1 protein that directly supports the water-oxidizing cluster in PSII (Ferreira et al., 2004; Fischer et al., 2015). Due to its role in water oxidation, this protein experiences high levels of oxidative damage and degradation. By impacting water oxidation, changes in redox such as sulfide inhibition of the water oxidizing complex in PSII (Miller and Bebout, 2004) and/or non-optimal light conditions lead to excessive energy (Klatt et al., 2015b) and influence the risk and magnitude of oxidative damage to D1 (Aro et al., 1993). Thus, cyanobacteria that experience such dynamic conditions may use another of the multiple *psbA* genes in their genomic repertoire under different light and oxygen regimes to mitigate oxidative damage (Schaefer and Golden, 1989; Campbell et al., 1999; Sicora et al., 2009; Gan et al., 2014; Cardona et al., 2015; Ho et al., 2016). *Geitlerinema* has two copies of *psbA* for OP under high oxygen and/or light levels (both *psbA3*; group 4), one gene for non-oxygen evolving PSII (*psbA1*; group 2), and one gene for microaerobic conditions (*psbA2*; group 3; **Figures 1** and **3**). We infer that these distinct photosynthetic genes reflect the adaptation of *Geitlerinema* to a variably lit, low-oxygen, and sulfidic lifestyle.

During high light conditions, when *Geitlerinema* conducts OP (Cohen et al., 1975a, 1986), the *psbA3* (group 4) genes are likely used to reduce photoinhibition and oxidative stress, as observed in *Synechococcus* sp. PCC 7942, *Synechocystis* sp. PCC6803, and *Thermosynechococcus elongatus* (Schaefer and Golden, 1989; Sicora et al., 2006; Kós et al., 2008; Sugiura et al., 2010). Other cyanobacteria synthesize identical D1 proteins from different genes at high light versus regular light conditions (Sugiura et al., 2010). Two copies of *psbA3* in its genome may equip *Geitlerinema* to continue OP in times of sufficient and/or high light, such as when mixed up into the epilimnion or in holomixis. Given that *psbA3* clusters with standard and high-light variant genes (**Figure 3**), it remains to be seen if one or both of *Geitlerinema*’s two copies of *psbA3* is cued to the different light intensities the organism may experience in its natural habitat.

The *psbA1* and *psbA2* genes likely provide *Geitlerinema* with the flexibility to conduct photosynthesis and nitrogen fixation under varying oxygen and/or sulfide concentrations. With sufficient light, the cyanobacterium may rely upon *psbA2* (group 3), which is keyed for microaerobic conditions. Changing redox environment promotes synthesis of *psbA*2-like variants (group 3) in *Thermosynechococcus elongatus* (Sugiura et al., 2012) and *Synechocystis* sp. PCC6803 (Sicora et al., 2004). However, light levels are lower than required for OP in the hypolimnion of Solar Lake, the prime habitat of *Geitlerinema*. Together with continuous sulfide exposure, these conditions often favor AP in cyanobacteria (Klatt et al., 2015a, 2016) and in green sulfur bacteria (Hanson et al., 2013; Findlay et al., 2015). Groups 2 and 3 *psbA* genes in the current study are in close chromosomal proximity to *sqr* genes (**Figure 3**), suggesting an intriguing but currently untested linkage between modified PSII and sulfide exposure. Alternative D1 proteins have been hypothesized to be directly involved in the transfer of electrons from donors other than water (Murray, 2012), such as in sulfide oxidation during nitrogen fixation (Becraft et al., 2015; Olsen et al., 2015). However, whether alternative D1 proteins are directly participating in this unusual photochemistry or serve to inactivate PSII in AP, remains to be tested.

Very little sulfide is required to limit OP in *Geitlerinema* (Cohen et al., 1986), and in the transition between oxygenic to AP, continuous exposure induces synthesis of proteins such as SQR (Arieli et al., 1991). SQR has a higher affinity for plastoquinone than PSII (Klatt et al., 2015a), thus in this induction period and with continual sulfide exposure, *Geitlerinema* may also reformulate its photosystem II machinery to reduce oxygen production. Finally, the *psbA1* (group 2) gene is also likely used to disable oxygen production during times of nitrogen fixation. Non-oxygen producing group 2 enzymes have been implicated as structural ‘placeholders’ that may allow oxygen-sensitive processes such as nitrogen fixation to occur (Sicora et al., 2009; Murray, 2012; Wegener et al., 2015). Further experiments targeting gene and protein expression will confirm the physiological roles of the variant *psbA* genes.

Variant *psbA* genes such as those observed in *Geitlerinema* likely permitted ancestral and potentially AP-capable cyanobacteria to meet metabolic requirements in dynamic physicochemical conditions. The importance of AP cyanobacteria and their metabolisms on Archean and Proterozoic oxygen levels is linked to the timing of OP. Mat-forming cyanobacteria in modern hypersaline and hot spring ecosystems likely face the same environmental challenges as their stromatolite-forming ancestors (Stal, 1995; Grotzinger and Knoll, 1999). As in modern systems, the metabolisms and mat-building lifestyles of ancestral cyanobacterial populations would have promoted light and redox dynamics on spatial (depth) and temporal (diel) scales (Canfield, 2005; Blank and Sánchez-Baracaldo, 2010; Lalonde and Konhauser, 2015; Sumner et al., 2015). Genomic and physiological studies on contemporary mat-forming cyanobacteria and their responses to changing environmental parameters, such as different versions of *psbA* keyed to light and/or oxygen levels, inform potential genetic and physiological strategies in ancient cyanobacteria. Given the long geologic history of variable and low atmospheric oxygen concentrations, the first appearance of alternative *psbA* in cyanobacteria is uncertain. The functional differences and potential heterotachy in these homologous genes dictate caution when evaluating their timing and evolutionary order. However, the basal arrangements of group 2 to group 3/4, and group 3 to group 4 in the phylogenetic tree hint at an ancestral development of water oxidation when atmospheric oxygen was low (Cardona et al., 2015). The perpetuation of diverse and evolutionarily old *psbA* genes in cyanobacterial genomes allow for efficient metabolic functioning regardless of oxygen levels/needs (Murray, 2012). These variant genes were likely retained in cyanobacterial genomes for oxygen-sensitive processes, such as nitrogen fixation, in an increasingly oxidizing environment.

### Nitrogen Acquisition Strategies in Variable Redox Conditions

*Geitlerinema* has a suite of genes for uptake of nitrogen (nitrate, nitrite, urea) as well as nitrogen fixation through a comprehensive nitrogenase gene suite (**Figures 1** and **2**). Sulfide-dependent AP was measured in select strains grown in nitrogen-replete media, including *Geitlerinema* sp. PCC 9228, *Coleofasciculus chthonoplastes*, and *Pseudanabaena* FS39, suggesting nitrate assimilation occurs under AP conditions (Cohen et al., 1986; Klatt et al., 2015a). *Geitlerinema* is also capable of AP-dependent nitrogen fixation (Belkin et al., 1982), potentially using sulfide to scavenge residual oxygen or donate electrons to nitrogenase (Stal, 2012).

Clues about the cyanobacterial transition from an anaerobic to aerobic lifestyle are apparent in regulatory genes for nitrogen acquisition. Due to its evolution under anoxia and strict requirement for anaerobic conditions, cyanobacterial diazotrophy in an increasingly oxidizing environment required new adaptations (Boyd et al., 2011; Stüeken et al., 2015). In the *nif* operon of *Geitlerinema* is *nifI_1_I_2_*, which encodes a signal transduction protein of the PII family that is present in diazotrophic archaea and select anaerobic bacteria, but has not been studied in cyanobacteria (Forchhammer, 2004; Boyd et al., 2015). In those organisms, NifI_1_I_2_ inhibits nitrogenase activity when the cell is no longer nitrogen limited (Kessler et al., 2001). *Geitlerinema*’s *nifHDK* gene suite is phylogenetically grouped with those from three other cyanobacterial genomes that also have *nifI_1_I_2_* in their nitrogenase operons (**Supplementary Figure S2**), and members of this cluster are adapted to low-oxygen and/or sulfidic conditions. *C. chthonoplastes* can continue to operate OP at low sulfide concentrations and is also capable of sulfide-dependent AP (Cohen et al., 1986). Similar to *Geitlerinema* and its *psbA1. Pleurocapsa* sp. PCC 7327 also has a *psbA* gene that codes for a rogue D1 protein typically used in anoxic conditions (Wegener et al., 2015).

### *Geitlerinema* Possesses Genetic Versatility for Sulfide Oxidation

Sulfide quinone reductase (SQR, encoded by *sqr*) oxidizes sulfide to elemental sulfur, and this process can be coupled with lithotrophic or phototrophic growth in bacteria and archaea or detoxification of sulfide in eukaryotes (Theissen et al., 2003; Marcia et al., 2010a). The genome of *Geitlerinema* sp. PCC 9228 holds two *sqr* operons. The first operon has the well-studied high-affinity *sqr1* (type I), which is translated after a few hours of exposure to micromolar levels of H_2_S (Oren and Padan, 1978) and enables *Geitlerinema* to grow by sulfide-based AP (Cohen et al., 1986). The transcriptional regulatory gene *arsR*, located downstream of *sqr1*, likely controls its expression (Nagy et al., 2014). Though lateral gene transfer has distributed various *sqr* types among bacteria, archaea, and eukarya (Theissen et al., 2003), type I *sqr* genes in cyanobacteria such as *Geitlerinema sqr1* form a distinct and well-supported cyanobacterial phylogenetic subclade within a bacterial clade (**Figure 4**). This cyanobacterial clade includes genes from AP-capable or sulfide-tolerant members such as *C. chthonoplastes. Synechocystis* sp. PCC6803, *H. halophytica*, and *Geitlerinema* sp. BBD (Cohen et al., 1986; Theissen et al., 2003; Marcia et al., 2010a; Nagy et al., 2014; Den Uyl et al., 2016). SQR is an evolutionarily ancient enzyme that was widespread in organisms during the Proterozoic (Theissen et al., 2003), during which the oceans had variable redox conditions including euxinia. Although the evolutionary history of *sqr* in cyanobacteria appears to be complex and remains unresolved, the *sqr1* gene is thought to be endogenous to cyanobacteria (Theissen et al., 2003). As a critical component of sulfide-based AP, this gene would have enabled ancestral cyanobacteria to thrive during the Proterozoic, when periods of photic zone euxinia would have favored AP and tempered oxygen production (Johnston et al., 2009). Whether the endogenous type I SQR may have permitted ancestral cyanobacteria to oxidize sulfide for energy, or cyanobacteria retooled this detoxifying enzyme for AP, is an open question.

The role of the second *sqr* operon in *Geitlerinema* is unknown. In addition to sulfide consumption through *sqr1*-based AP (Oren and Padan, 1978), *Geitlerinema* has a second sulfide donation site on the immediate donor side of PSI, which is not inducible (i.e., does not require protein synthesis) and does not significantly contribute to proton translocation (Shahak et al., 1987). However, this response operates at even higher concentrations of sulfide (K_m_ in the mM range) without saturation (Shahak et al., 1987; Arieli et al., 1991). The enzyme mediating this response to high sulfide is unknown, thus it could be encoded by this *sqr2*. Only 10 of the 44 cyanobacterial genomes and genomic bins examined in this study (including *Geitlerinema* sp. PCC 9228) have more than one version of *sqr*. Seven genomes in this subset, such as *Synechocystis* sp. PCC6803 (Nagy et al., 2014), each have two versions *sqr*, one of which is phylogenetically similar to *sqr1*, and the other a eukaryotic homolog (type II). Only three genomes, all of them *Geitlerinema* species, have genes phylogenetically similar to *sqr2* (type VI) as well as an *sqr1*-like version. The sulfide physiology of *Geitlerinema* sp. PCC7105 is unknown, but *Geitlerinema* sp. BBD is a known sulfide-resistant photosynthetic cyanobacterium (Den Uyl et al., 2016). Green sulfur bacteria, thiotrophic proteobacteria, and members of Aquificaceae have multiple versions of *sqr* or homologs with a range of sulfide affinities. *Aquifex aeolicus* expresses its types I and VI *sqr*, similar to *Geitlerinema’s sqr1* and *sqr2*, even when not growing thiotrophically (Marcia et al., 2010a,b). On the other hand, *Chlorobium* is capable of sulfide-dependent growth at mM H_2_S by using its type VI *sqr*, and other versions are used at lower sulfide concentrations (Chan et al., 2009). Phylogenetic clustering of *sqr2* with green sulfur bacterial *sqrs*, including one used at high sulfide levels, supports a similar role in *Geitlerinema*. Like *sqr1. sqr2* also has a transcriptional regulatory gene *arsR* in the operon. These observations raise the possibility that *Geitlerinema* is capable of growing phototrophically on different sulfide levels through its *sqr1* and *sqr2*. Our results also suggest that such versatility was likely achieved through a combination of evolutionary processes including vertical descent within ancestral cyanobacteria (*sqr1*, type I clade) as well as lateral transfer from other groups (*sqr2*, type VI clade). When these genes became part of the AP-cyanobacterial repertoire is uncertain.

Chromosomal examination of cyanobacterial *sqr* and groups 2 and 3 *psbA* genes underscores a potential relationship between sulfide exposure and reformulation of the water-oxidizing complex in periods of AP and/or OP. Of the evaluated cyanobacterial genomes with *sqr*, 15 of the genomes have *sqr* types I, II, or VI in close genetic proximity to low-oxygen or anaerobic *psbA* genes. These genomes include metagenomic bins from Middle Island Sinkhole, where sulfide-based AP has been demonstrated (Voorhies et al., 2012), two other species of *Geitlerinema*, four *Cyanothece* species, and two *Leptolyngbya* species, among others. Because of variable contig lengths, it is unknown if *psbA1* and/or *psbA2* of *Geitlerinema* sp. PCC 9228 are in proximity to one or both of its *sqr* genes. However, the small intergenetic spaces between anoxygenic/micro-oxygenic *psbA* varieties and *sqr* in the other genomes hints at potential linked transcriptional regulation of these genes. Additionally, of the cyanobacterial *sqr* genes, 27 were in close proximity to *arsR*-like transcriptional regulators. These genes and conditions of their expression are an attractive target for further studies.

### Potential Trace Metal Oxidation

Different versions of *sqr* in *Geitlerinema* sp. PCC 9228 may also be linked to trace metal metabolism and resistance. In close proximity to *sqr2* there are genes for *arsC. arsB. arsR*, and glutathione (GST) *S*-transferase, and on other contigs there are genes for arsenite transporting ATP-ases *arsA*, another *arsB*, a methyltransferase similar to arsenite methylator *arsM*, and a chromate transporter similar to a cyanobacterial arsenite importer. These genes are involved in arsenate reduction, arsenite transport, transcriptional regulation, and mediation of arsenic resistance (Oden et al., 1994; Mukhopadhyay et al., 2002; Cameron and Pakrasi, 2010; Slyemi and Bonnefoy, 2011). *Synechocystis* sp. PCC6803 uses an *arsR* gene to regulate genomic *arsBHC* expression during arsenic exposure (López-Maury et al., 2003), and is able to grow in mM concentrations of arsenite and arsenate (Sánchez-Riego et al., 2014). An *arsR*-like transcriptional regulator is also adjacent to *sqr1* in *Geitlerinema* (Bronstein et al., 2000), and the proximity of *arsR* to *sqr2* suggests a similar regulatory role. Arsenic resistance genes are widely distributed among bacteria and archaea, and may be found in environments that do not have measurable arsenite (Mukhopadhyay et al., 2002; Oremland and Stolz, 2005). The hydrology of the habitat of *Geitlerinema*, Solar Lake, is driven primarily by seawater seepage through the sand bar, with minor meteoric input (Cohen et al., 1977a). Usual sources of arsenic, such as hydrothermal hot springs in hypersaline environments or weathering of arsenic-rich clays (Oremland et al., 2009), are absent from this system. As such, these genes may have been inherited from ancestral cyanobacteria inhabiting a metal-rich environment (Oremland et al., 2009), be used to detoxify other metals such as antimony (Nagy et al., 2014), or cope with reactive oxygen species and oxidative stress (Latifi et al., 2009; Takahashi et al., 2011).

The proximity of the *sqr2* to arsenic-related genes (*arsRBC*, glutathione *S*-transferase), taken together with experimental results from related organisms and genes, hints at an unexplored potential metabolism in *Geitlerinema*: AP using arsenite as the electron donor, as in anoxygenic bacteria (Kulp et al., 2008; Hoeft et al., 2010; Edwardson et al., 2014). *Oscillatoria*-like cyanobacterial biofilms in an arsenic-rich hot spring, and *Synechocystis* sp. PCC 6803, can perform light-dependent arsenite oxidation (Kulp et al., 2008; Nagy et al., 2014). In *Synechocystis* sp. PCC 6803, the same *sqr* and *arsR* genes from its plasmid *sqr* operon, which enable light-dependent sulfide oxidation, are expressed during arsenite (As(III)) oxidation in the light (Nagy et al., 2014). The cyanobacterium imports arsenite through a chromate transporter (*suoT*, on the same plasmid operon), stores mM arsenite intracellularly without detriment, and exports excess arsenite through *arsB* (Nagy et al., 2014). The arrangement of *Geitlerinema’s* arsenic-related genes on its *sqr2* operon is similar to the plasmid *sqr* operon of *Synechocystis* sp. PCC 6803. Arsenite oxidation has been linked to electron transfer to quinones (Jiang et al., 2009) and energetics support the potential of cyanobacterial plastoquinone as an arsenite oxidant (Nagy et al., 2014), but this pathway has not been explored in cyanobacteria. The co-transcription of *sqr1* in *Synechocystis* with arsenite uptake genes during arsenite exposure (Nagy et al., 2014), and the well-characterized electron-stripping mechanism of *sqr1* on sulfide (Arieli et al., 1994), hints at a role for *sqr* in arsenite oxidation.

Arsenite-dependent cyanobacterial AP is intriguing due to the role of arsenite-based primary production on ancient Earth. Arsenic was likely more abundant on Earth’s surface during the Archean than present (Oremland et al., 2009). In anoxic marine basins that dominated the biosphere 2.7 Ga ago, arsenite-dependent microbial autotrophy putatively cycled nitrogen, carbon, and arsenic (Sforna et al., 2014). This metabolism continues in modern hypersaline hot springs and subsurface aquifers that have elevated arsenic levels (Oremland and Stolz, 2005; Kulp et al., 2008; Hoeft et al., 2010). The genome of *Geitlerinema* lacks proteobacterial arsenite-oxidizing genes, and instead has genes similar to those used for light-dependent arsenite oxidation in *Synechocystis* sp. PCC 6803 (Nagy et al., 2014). Verifying arsenite-dependent AP in cyanobacteria, and conclusively linking the *sqr2* gene in *Geitlerienema* to that process, would clarify the potential role of anoxygenic cyanobacteria in arsenic cycling in both modern and ancient ecosystems.

In summary, analysis of the genome of *Geitlerinema* sp. PCC9228 complements prior physiology studies by providing the genetic foundation for its metabolisms of nitrogen fixation, facultative OP, and sulfide-based AP. We find multiple versions of *psbA*, encoding a key protein for water oxidation, which may enable a sensitive response to varying conditions of light, oxygen and sulfide. Nitrogen fixation is linked to oxygen level and production via the *nifI_1_I_2_* regulator in the *nif* operon and via non-oxygen producing *psbA*, respectively. Multiple versions of *sqr* likely address a range of sulfide concentrations and may also be linked to responses to metals and oxidative stress and perhaps even arsenite oxidation. Aerobic versatility encoded in the genome of *Geitlerinema*, coupled with diazotrophic regulation and concentration-specific sulfide responses, permit *Geitlerinema* to thrive in periodic sulfidic, microoxic, and poorly lit conditions of Solar Lake (Cohen et al., 1975a). Such dynamic geochemical conditions likely also challenged cyanobacteria during variable sulfide and oxygen levels of the Archean and Proterozoic (Satkoski et al., 2015; Sperling et al., 2015). This study of *Geitlerinema* and its unique gene assemblage addresses both the ambiguous role of Archean cyanobacteria in oxygen production/mitigation prior to the development of OP and the Great Oxidation Event, as well as the contributions of AP cyanobacteria to Proterozoic biogeochemistry. The phylogeny and diversity of genes responsible for metabolic versatility in *Geitlerinema* suggest a blend of genetic strategies for the anoxic early environment—such as methanogen-like modulation of nitrogen fixation and non-water oxidizing photosynthetic proteins—with post-oxidation strategies such as specific photosynthetic proteins for micro-oxic as well as oxic conditions, and different SQRs for fluctuating sulfide concentrations. Phototrophs capable of versatile AP/OP, such as *Geitlerinema*, would have had the advantage over organisms metabolically limited to either oxic or sulfidic conditions. Their continuous photosynthesis likely supported other microorganisms with fixed nitrogen and carbon, sulfide removal, and intermittent oxygen production. Furthermore, conditional production of oxygen at variable concentrations would have had strong influences on the structure and metabolic needs of their associated microbial communities through development of oxygen refugia and/or oases. Further research into *Geitlerinema’s* growth and transcriptional regulation will uncover the fine-tuned response of AP/OP cyanobacteria to changing redox conditions. In turn, we can relate the scope of these dual metabolisms and their modern physiologies to their ancestors’ impacts on ecology and geochemistry as Earth slowly and discontinuously became oxygenated.

## Author Contributions

SG designed the study and conducted phylogenetic analyses. GD supervised the analyses. SG and GD contributed to the manuscript.

## Conflict of Interest Statement

The authors declare that the research was conducted in the absence of any commercial or financial relationships that could be construed as a potential conflict of interest.

## Supplementary Material

**FIGURE S1 | Emergent self-organized map (ESOM) of *Geitlerinema* sp. PCC 9228 genomic contigs, clustered on the basis of tetranucleotide frequency.**

**FIGURE S2 | Phylogenetic tree of *nifHDK*.** The clade with *Geitlerinema* sp. PCC 9228 includes three other cyanobacterial genomes that also have *nifI*_1_*I*_2_. The outgroup is the homologous *chlLNB* gene suite from *Halothece halophytica*.

**FIGURE S3 | Full phylogenetic tree of *psbA* (with non-collapsed clades).** Genes are colored by groups 1–4 modeled after (Cardona et al., 2015). The outgroup is a *Gloeobacter kilaueensis JS psbA* that is most like *psbD* and is unable to oxidize water. Purple indicates group 1, red genes belong to group 2, orange genes are members of group 3, and blue standard psbAs belong to group 4. Genetic proximity to types I (green circle), II (maroon squares), and VI (blue stars) *sqr* genes are indicated. Fourteen *psbA* genes are five or fewer genes from sqr, and the largest gap is 19 genes. *Geitlerinema* sp. PCC 9228 has members of groups 2–4.

**FIGURE S4 | Schematic of *sqr* operons in *Geitlerinema* sp. PCC 9228.** **(A)** Canonical *sqr* (type I) with regulatory gene *arsR* collocated downstream. **(B)** Novel *sqr* (type VI) with *arsR*, arsenite transport gene *arsB*, and arsenate reductase *arsC* all downstream, and glutathione *S*-transferase and a multidrug efflux pump are upstream.

**FIGURE S5 | Full phylogenetic tree of sulfide quinone reductase (*sqr*).** Genes are colored by types I–VI modeled after (Marcia et al., 2010a). Bacterial FCSD (yellow) includes representatives of flavocytochrome c:sulfide dehydrogenase. Green sulfur bacteria have *sqr* belonging to types III (orange), V (light blue), IV (dark green), and VI (blue). Cyanobacterial *sqr* are limited to types I (light green), II (red), and VI. Proximity to *psbA* versions on the same contig (see **Figure 3**; **Supplementary Figure S3**) are indicated with red circles (group 2) and brown stars (group 3). No *sqr* genes in this analysis were genetically proximal to groups 1 or 4 *psbA* genes. *Geitlerinema* sp. PCC 9228 has types VI and I *sqr*.
