# Supplementary figures and images for: Photosynthetic Versatility in the Genome of Geitlerinema sp. PCC 9228 (Formerly Oscillatoria limnetica ‘Solar Lake’), a Model Anoxygenic Photosynthetic Cyanobacterium
Source: Front Microbiol. 2016 Oct 13;7:1546. doi: 10.3389/fmicb.2016.01546 (PMC5061849; doi:10.3389/fmicb.2016.01546)

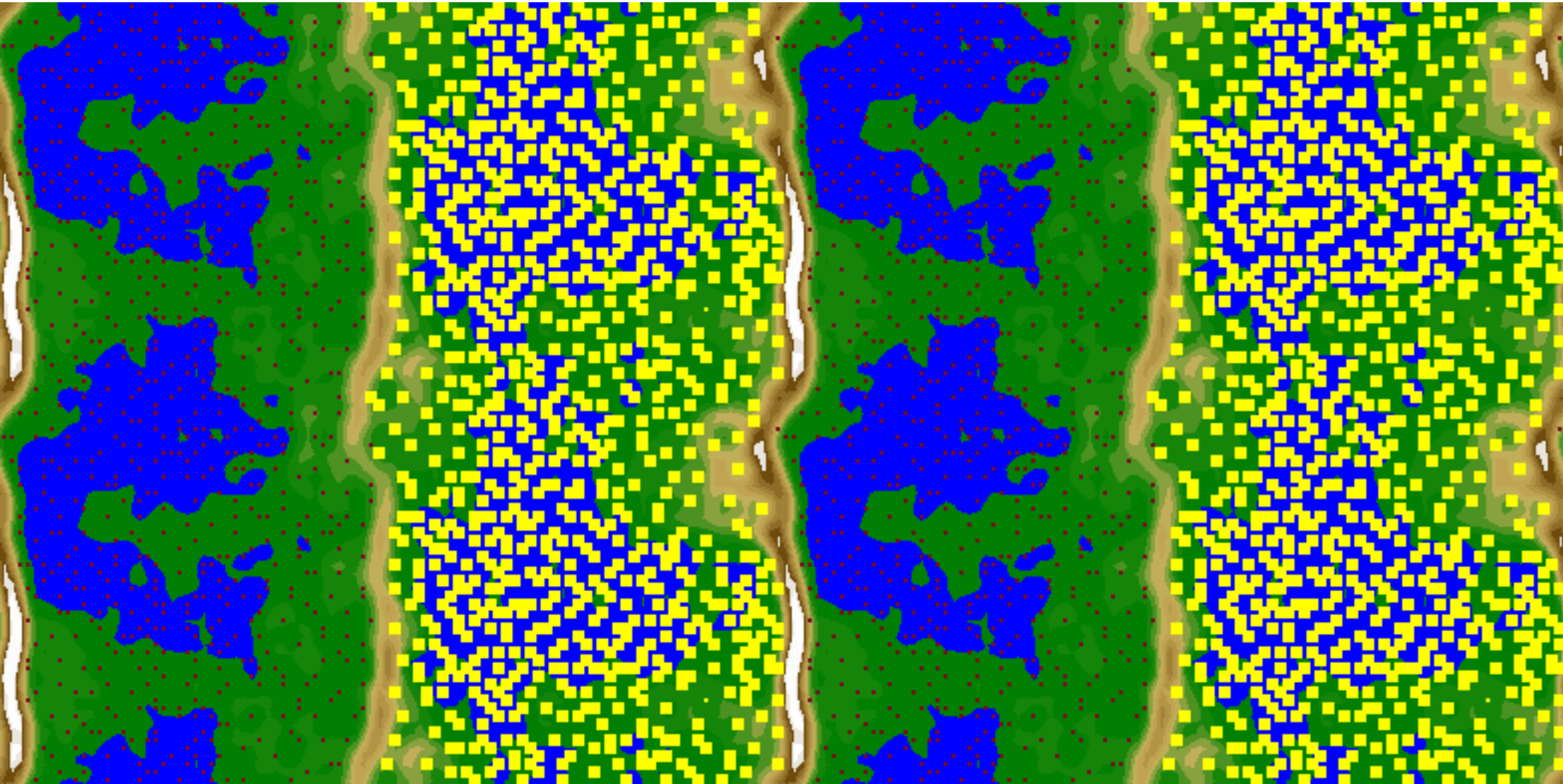

Supplement: Supplementary file 1 [file Image_1.PDF]

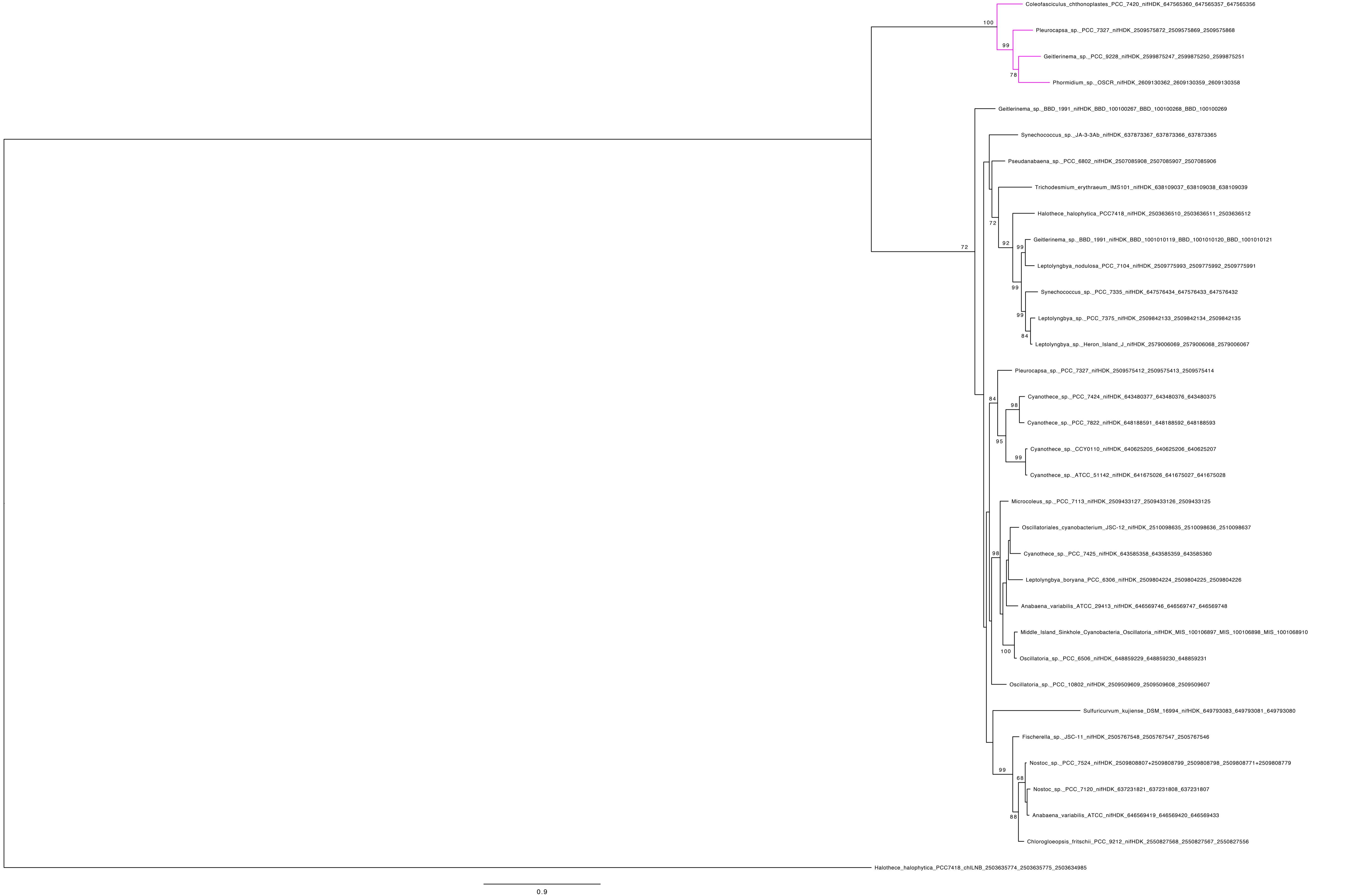

Supplement: Supplementary file 2 [file Image_2.PDF]

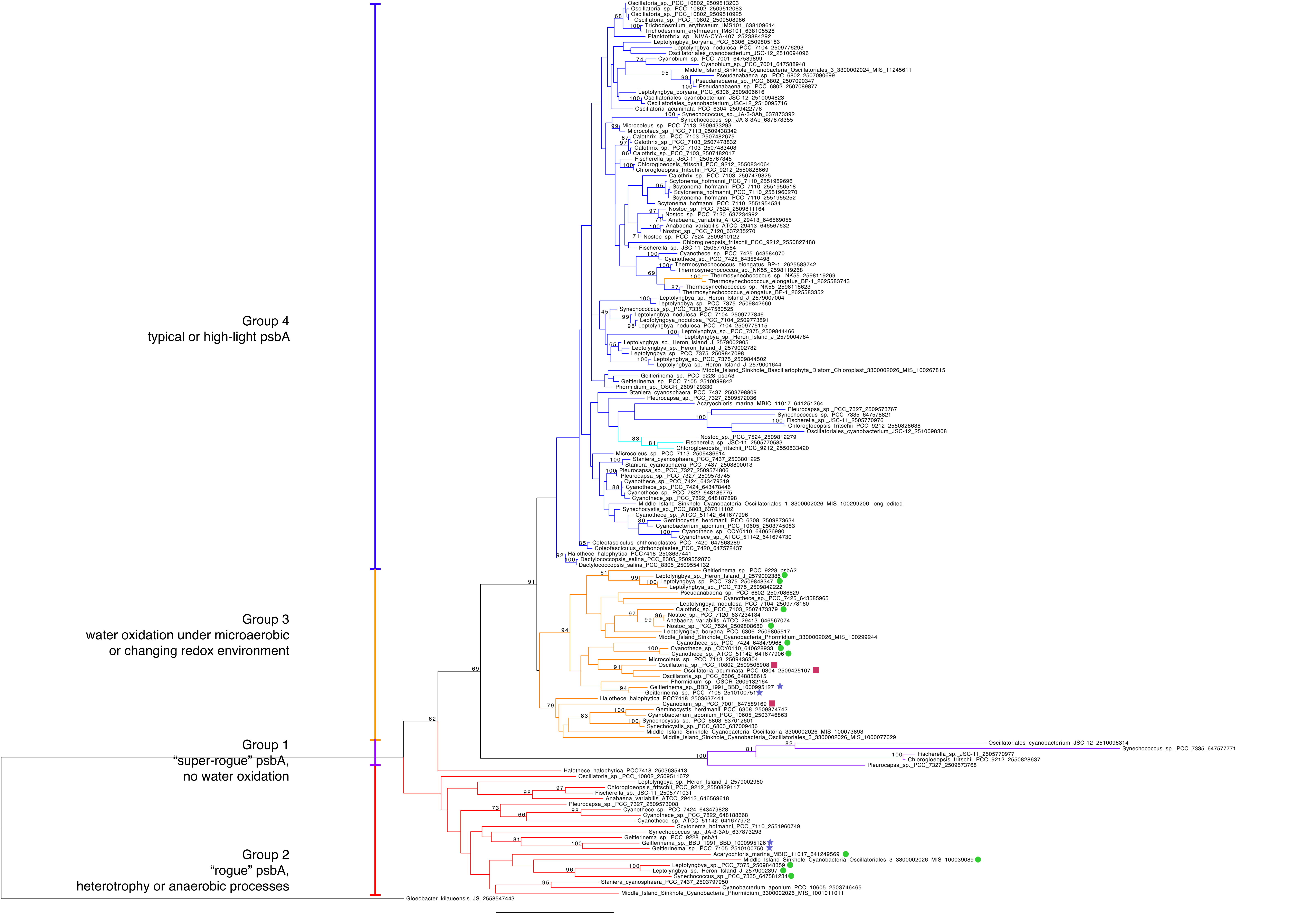

Supplement: Supplementary file 3 [file Image_3.PDF]

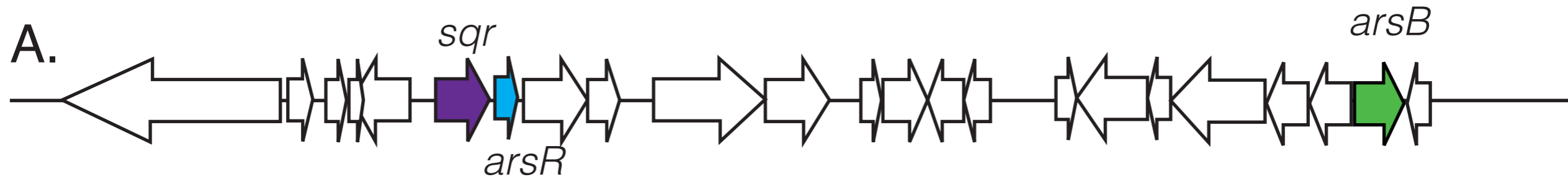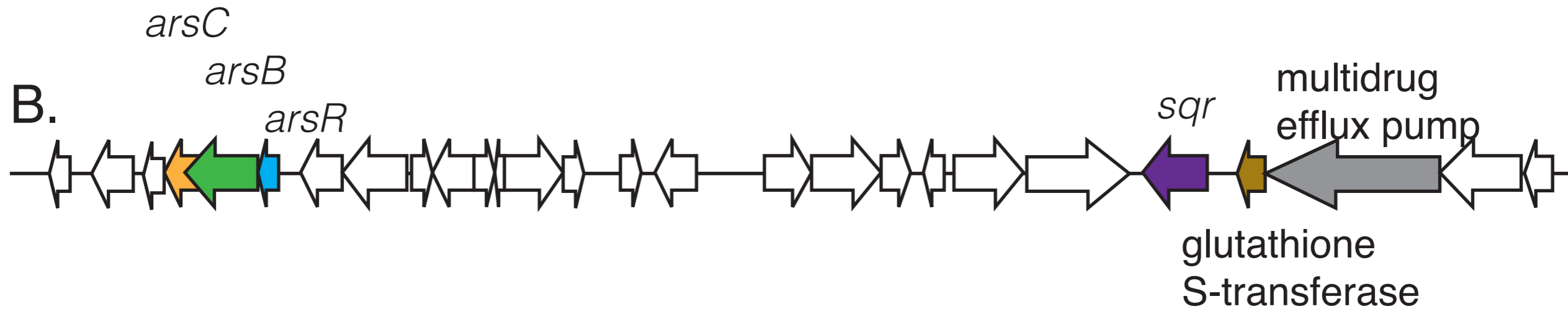

Supplement: Supplementary file 4 [file Image_4.PDF]

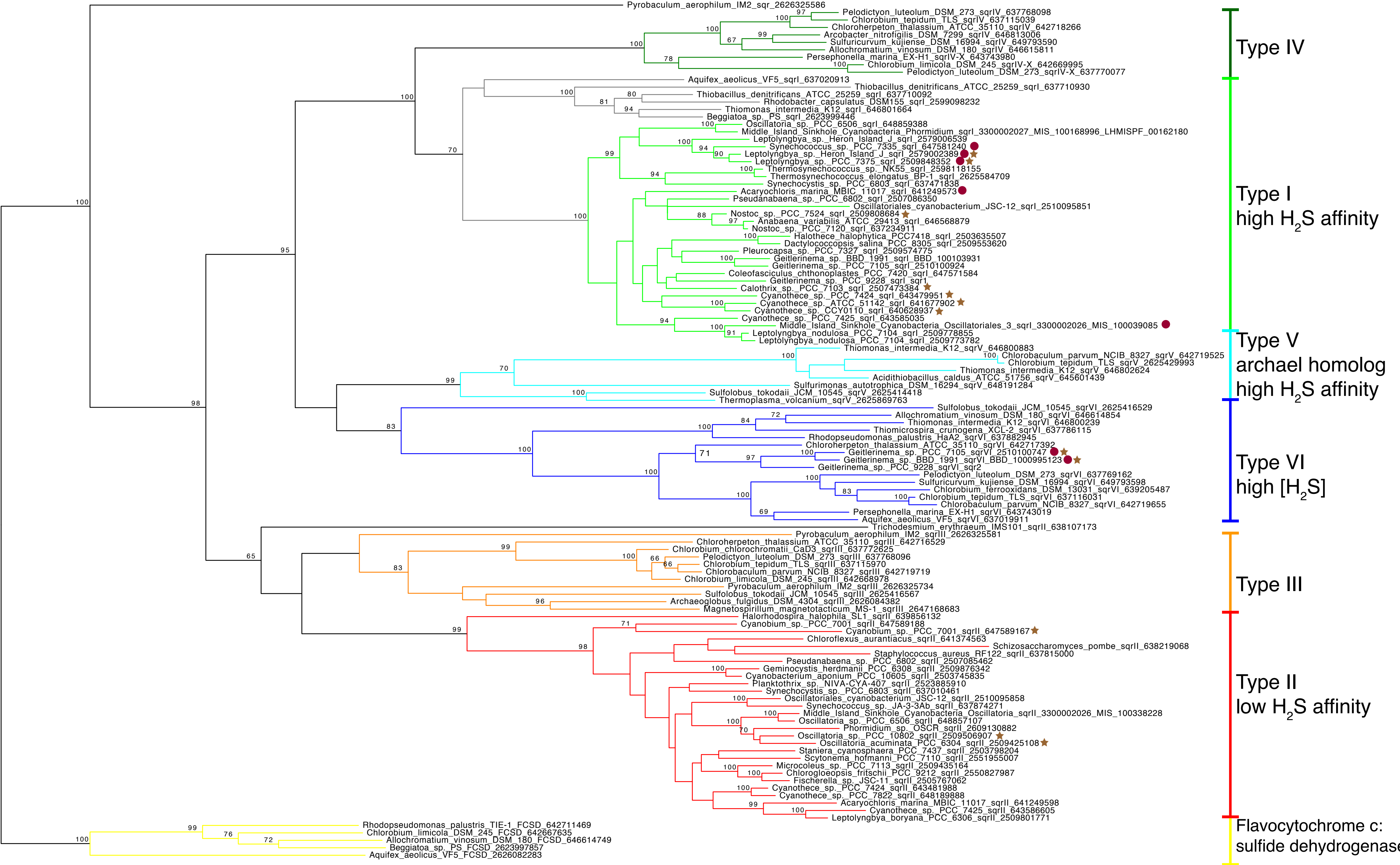

0.4

Supplement: Supplementary file 5 [file Image_5.PDF]
